# From Screening to Outcomes: Fourteen-Year Hospital-Wide Surveillance of Alert Pathogens and Antimicrobial Use in a Paediatric Tertiary Hospital

**DOI:** 10.3390/antibiotics15020118

**Published:** 2026-01-26

**Authors:** Aleksandra Tukendorf, Julia Burzyńska, Katarzyna Semczuk, Ryszard Sot, Katarzyna Dzierżanowska-Fangrat

**Affiliations:** 1 Department of Clinical Microbiology and Immunology, Hospital Infection Control Team, The Children’s Memorial Health Institute, Aleja Dzieci Polskich 20, 04-730 Warsaw, Poland; a.tukendorf@ipczd.pl (A.T.); j.burzynska@ipczd.pl (J.B.); k.semczuk@ipczd.pl (K.S.); 2 Department of Hospital Pharmacy, The Children’s Memorial Health Institute, 04-730 Warsaw, Poland; r.sot@ipczd.pl

**Keywords:** infection prevention and control, hand hygiene, admission screening, antimicrobial stewardship, paediatrics, antibiotic consumption

## Abstract

**Background/Objectives**: Infection prevention and control (IPC) programs combine pathogen-targeted measures (e.g., admission screening) with hospital-wide standard precautions (e.g., hand hygiene, HH). We assessed temporal associations between screening, HH, antimicrobial stewardship (AMS), and hospital-level outcomes in a tertiary paediatric hospital. **Methods**: This study was a retrospective hospital-wide ecological time-series at the Children’s Memorial Health Institute. Annual aggregate data: 2011–2024 for screening, colonisation, and healthcare-associated infections (HAIs) with alert pathogens; 2016–2024 for antibiotic consumption (ATC J01, systemic antibacterials). Process indicators: number of screening tests and alcohol-based hand rub (ABHR) consumption per 1000 patient-days (PD). Outcomes: colonisations/HAIs per 1000 PD and defined daily doses (DDD) per 1000 PD overall and by class. Trends used linear regression and Spearman’s rank correlation. **Results**: Screening intensity increased from 39 to 150/1000 PD (slope +8.3/year; R^2^ = 0.90; *p* < 0.001). Detected colonisation rose (2.5 → peak 8.05/1000 PD in 2023; slope +0.39; R^2^ = 0.81; *p* < 0.001), while multidrug-resistant-organism (MDRO)-attributable HAIs remained low/stable (0.27–0.62/1000 PD; slope −0.014; *p* = 0.023). ABHR consumption increased from 26.1 to 78.0 L/1000 PD in 2020 (*p* < 0.001) and partially normalised to 60.0 in 2024 (>2 × baseline). Overall ATC J01 showed no long-term linear trend (~278–356 DDD/1000 PD; +2.57/year; *p* = 0.46), but class mix shifted: carbapenems, fluoroquinolones, and amoxicillin–clavulanate decreased; third/fourth-generation cephalosporins, piperacillin/tazobactam, and glycopeptides increased. **Conclusions**: In this tertiary paediatric setting, expansion of risk-based admission screening and sustained implementation of horizontal IPC measures were accompanied by increased detection of colonisation with alert pathogens, while MDRO-attributable HAIs remained low and stable at the hospital level. Over the same period, AMS activity coincided with a redistribution in antibiotic class use without a clear long-term reduction in total antibiotic consumption. These hospital-level findings are descriptive and hypothesis-generating; causal inference is limited by the ecological study design, and the heterogeneous, multispecialty structure of a tertiary paediatric centre.

## 1. Introduction

Healthcare-associated infections (HAIs) remain a major patient-safety threat and a driver of antimicrobial resistance (AMR) in Europe [1]. The ECDC estimates that more than 3.5 million HAIs occur each year in the EU/EEA, contributing to over 90,000 deaths and around 2.5 million DALYs [1]. Recent ECDC point prevalence survey (PPS) results further indicate that approximately 4.3 million hospital patients in the EU/EEA acquire at least one HAI annually [2].

The rising burden of antibiotic-resistant infections amplifies this challenge. Updated ECDC estimates for 2016–2020 show a substantial and, for some pathogen–resistance combinations, increasing health burden of infections caused by antibiotic-resistant bacteria across the EU/EEA [3].

Robust infection prevention and control (IPC) programmes, particularly hand hygiene (HH) implemented through the WHO multimodal improvement strategy, are core to HAI prevention. WHO reports that effective IPC interventions can reduce HAIs and AMR by 35–70% and are cost-saving across income settings [4]. The WHO ‘My 5 Moments for Hand Hygiene’ approach defines key moments for HH and provides field-tested implementation tools for healthcare facilities [5].

In parallel, risk-based admission screening for carriers of highly resistant organisms is recommended to support timely isolation and other transmission-based precautions. The ECDC guidance specifically advises pre-emptive isolation and screening upon admission for patients at increased risk of carbapenem-resistant Enterobacterales (CRE) colonisation [6].

Antimicrobial stewardship (AMS) complements IPC by optimising antimicrobial exposure at hospital level. WHO practical guidance describes commonly used stewardship interventions and their evidence base, supporting tailored implementation in different resource settings [7]. Collectively, IPC and AMS aim to prevent transmission, maintain low HAI rates, and reduce the selective pressure associated with broad-spectrum antibiotic use [7,8].

Against this backdrop, we conducted a 14-year hospital-wide surveillance study at a tertiary paediatric centre (2011–2024) to examine the temporal relationships between (i) admission screening intensity; (ii) colonisation and hospital infections due to alert pathogens; (iii) hand-hygiene activity; and (iv) antimicrobial use (overall and by class). We hypothesised that the escalation of screening and sustained IPC/AMS activity could coincide with higher colonisation detection yet stable/low HAI rates, alongside favourable shifts in antibiotic class mix.

## 2. Results

Over the study period, the hospital activity and care structure evolved gradually. The annual number of hospital admissions increased, while the estimated mean length of stay decreased steadily from 4.01 days in 2011 to 2.36 days in 2024, indicating a marked shortening of average hospitalisation duration. In parallel, combined PICU and NICU activity accounted for approximately 11–17% of total hospital patient-days, with year-to-year variation but no abrupt shifts in overall care intensity (Supplementary Table S2).

Against this background, we analysed annual hospital-level trends in admission screening activity, colonisation and HAIs with alert pathogens, HH activity, and antimicrobial consumption.

As a process indicator of the hospital-level admission screening strategy for alert pathogens, the number of screening tests performed at CMHI increased steadily between 2011 and 2024. At the start of the period (2011) an average of 39 tests/1000 patient-days were performed, rising to 150/1000 patient-days in 2024—almost a four-fold increase. The upward trend was strong and significant, corresponding to an average annual increase of +8.3 tests/1000 patient-days (R^2^ = 0.90; *p* < 0.001; ρ = 0.94; *p* < 0.001). This increase coincided with the progressive expansion of the screening eligibility criteria over time, in the context of concurrent changes in hospital throughput and length of stay.

As the screening intensified, the detection of colonisation with alert pathogens increased. Colonisation rose from 2.5/1000 patient-days in 2011 to values above 7/1000 patient-days after 2018, peaking at 8.05/1000 patient-days in 2023. The mean annual increase was +0.39/1000 patient-days (R^2^ = 0.81; *p* < 0.001; ρ = 0.81; *p* = 0.005).

**Figure 1:**
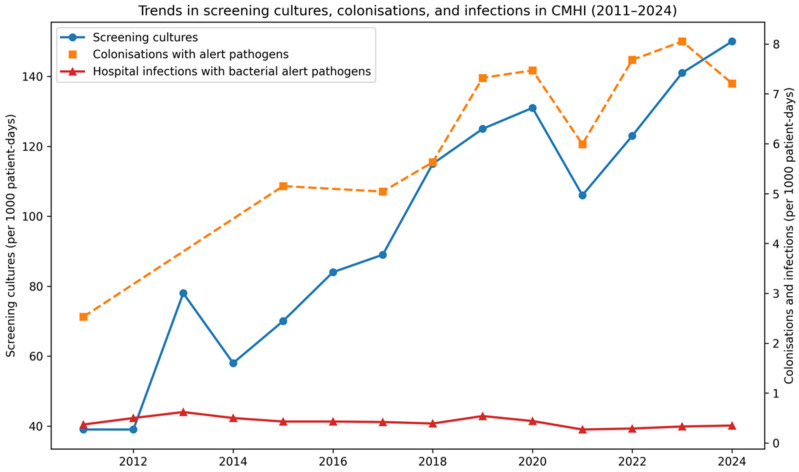
Trends in screening cultures, colonisation with bacterial alert pathogens, and HAIs with bacterial alert pathogens at CMHI, 2011–2024.

In contrast to colonisation, the incidence of hospital infections caused by bacterial alert pathogens remained low and stable (0.27–0.62/1000 patient-days). Over the entire period, there was a slight downward tendency of −0.014/1000 patient-days per year (R^2^ = 0.36; *p* = 0.023; ρ = −0.59; *p* = 0.026). Figure 1 shows the relationship between the screening cultures, colonisation, and HAIs caused by bacterial alert pathogens.

Among the alert pathogens analysed, ESBL–producing Enterobacterales were the most frequently detected, followed by other multidrug-resistant organisms with distinct temporal patterns of colonisation and infection. ESBL colonisation increased systematically, from 2.3/1000 patient-days in 2011 to > 6/1000 patient-days after 2021, peaking in 2023 (6.4/1000 patient-days). The trend was significant, with a mean annual increase of +0.34/1000 patient-days (R^2^ = 0.79; *p* < 0.001; ρ = 0.82; *p* = 0.002). Hospital ESBL infections remained low and stable (0.24–0.58/1000 patient-days) with no significant change over time (*p* = 0.21).

Colonisation with CPE was not observed until 2017. The first cases appeared in 2018 (0.01/1000 patient-days), increasing to 0.10–0.16/1000 patient-days in subsequent years—an increasing trend in 2018–2024 (average +0.02/1000 patient-days per year; *p* = 0.01). Only one hospital CPE infection occurred by the end of the study period (2024).

Colonisation with MRSA remained stable at a low level (0.2–0.5/1000 patient-days), with no significant trend (*p* = 0.47). Hospital MRSA infections were rare (0.01–0.05/1000 patient-days) and did not change over time (*p* = 0.68).

VRE colonisation occurred sporadically early on (<0.2/1000 patient-days), then increased after 2015, reaching 0.4–0.8/1000 patient-days in 2018–2020, and peaking in 2023 (0.91/1000 patient-days). The upward trend was significant: +0.06/1000 patient-days per year (R^2^ = 0.63; *p* = 0.008; ρ = 0.71; *p* = 0.01). Hospital VRE infections were sporadic (0.01–0.04/1000 patient-days), with no significant trend (*p* = 0.34).

Colonisation with CAR-R remained very low (0.01–0.18/1000 patient-days), without a significant trend (*p* = 0.41). Hospital CAR-R infections were more frequent in the early years (0.06–0.08/1000 patient-days in 2011–2015) but declined significantly after 2020, by −0.006/1000 patient-days per year (R^2^ = 0.48; *p* = 0.03; ρ = −0.62; *p* = 0.02), reaching a minimum of 0.01/1000 patient-days in 2023 and 2024.

**Figure 2:**
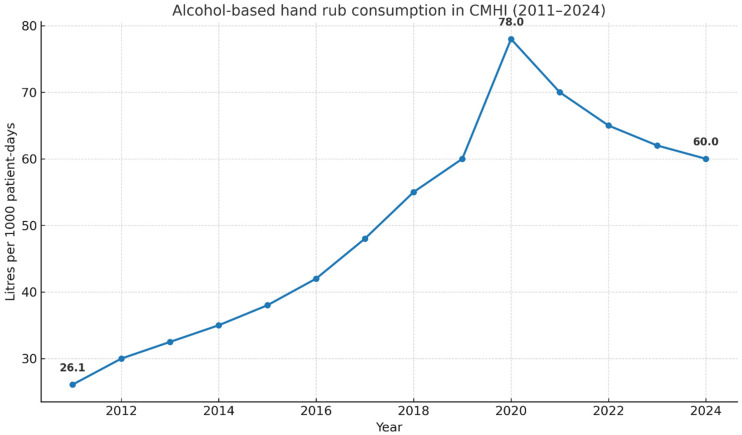
Annual consumption of ABHR at CMHI, 2011–2024 (L per 1000 patient-days).

As a process indicator of hand-hygiene activity within a long-standing hospital-wide infection prevention and control programme, ABHR consumption increased from 26.1 L/1000 patient-days in 2011 to 78.0 L/1000 patient-days in 2020 (average +5.8 L/1000 patient-days per year; *p* < 0.001). The highest values were recorded in the first pandemic year, with a subsequent decrease to 60.0 L/1000 patient-days in 2024 (average −4.5 L/1000 patient-days per year); however, this remained more than twice the baseline level (*p* < 0.001) (Figure 2).

**Figure 3:**
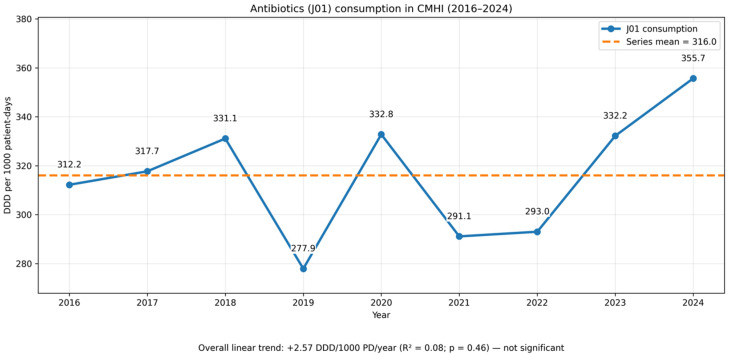
Overall antibiotic consumption (ATC J01) in CMHI, 2016–2024 (DDD per 1000 patient-days). Values fluctuated between ~278 and 356 with no significant overall linear trend (+2.57/year; R^2^ = 0.08; *p* = 0.46). Dashed line shows the series mean.

The overall antibiotic consumption (ATC J01) at CMHI in 2016–2024 ranged from ~278 to 356 DDD/1000 patient-days and showed no significant trend in the linear analysis (+2.57 DDD/1000 patient-days per year; R^2^ = 0.08; *p* = 0.46) or in Spearman’s test (ρ = 0.35; *p* = 0.356). After a period of lower use in 2019–2022, an increase was observed in 2023–2024 (up to 355.69 DDD/1000 patient-days), which was significant when restricted to 2021–2024 (+23.29/year; R^2^ = 0.91; *p* = 0.044) (Figure 3).

**Figure 4:**
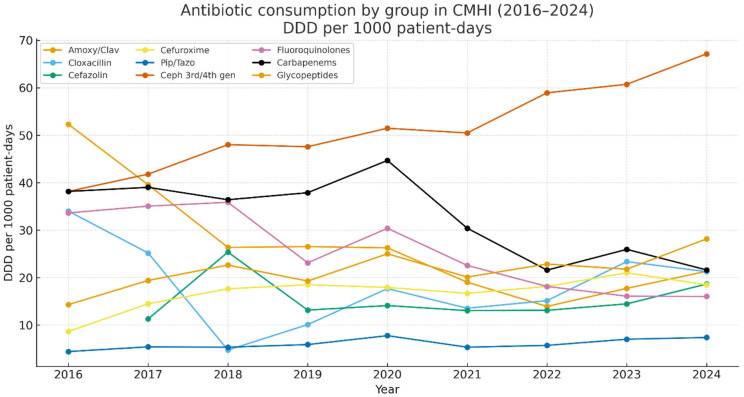
Shifts in antibiotic class use at CMHI, 2016–2024 (DDD per 1000 patient-days). Amoxicillin–clavulanate, fluoroquinolones, and carbapenems decreased; third/fourth-generation cephalosporins, cefuroxime, piperacillin/tazobactam, and glycopeptides increased.

When antibiotic consumption was analysed by individual classes, a clear redistribution of use across antibiotic groups was observed over time (Figure 4). The use of several commonly prescribed broad-spectrum agents, including amoxicillin–clavulanate, fluoroquinolones, and carbapenems, decreased substantially. Amoxicillin–clavulanate declined from 52.32 to 21.34 DDD/1000 patient-days (−3.70/year; 95% CI −5.44 to −1.95; R^2^ = 0.71; *p* = 0.004; ρ = −0.87; *p* = 0.002), fluoroquinolones from 33.64 to 16.04 DDD/1000 patient-days (−2.72/year; 95% CI −3.63 to −1.81; R^2^ = 0.83; *p* < 0.001; ρ = −0.92; *p* < 0.001), and carbapenems from 38.17 to 21.63 DDD/1000 patient-days (−2.38/year; 95% CI −3.77 to –0.98; R^2^ = 0.61; *p* = 0.012; ρ = −0.75; *p* = 0.020).

In contrast, the use of second- to fourth-generation cephalosporins, piperacillin/tazobactam, and glycopeptides increased. Third- and fourth-generation cephalosporins rose from 38.17 to 67.16 DDD/1000 patient-days (+3.29/year; 95% CI +2.72 to +3.86; R^2^ = 0.95; *p* < 0.001; ρ = 0.97; *p* < 0.001), while cefuroxime increased from 8.66 to 18.50 DDD/1000 patient-days (+0.97/year; 95% CI +0.34 to +1.59; R^2^ = 0.57; *p* = 0.019; ρ = 0.73; *p* = 0.025). Piperacillin/tazobactam showed a more moderate increase, from 4.43 to 7.39 DDD/1000 patient-days (+0.28/year; 95% CI +0.07 to +0.50; R^2^ = 0.48; *p* = 0.038; ρ = 0.64; *p* = 0.061), as did glycopeptides, which rose from 14.32 to 28.17 DDD/1000 patient-days (+1.06/year; 95% CI +0.35 to +1.77; R^2^ = 0.55; *p* = 0.022; ρ = 0.70; *p* = 0.036).

No significant trends were observed for cefazolin (data available from 2017; *p* = 0.95) or cloxacillin (*p* = 0.67). As a composite indicator of broad-spectrum antibiotic pressure, the combined use of carbapenems and fluoroquinolones decreased by approximately 48% between 2016 and 2024.

## 3. Discussion

A robust IPC system should primarily rest on the effective surveillance of HAIs and on trained personnel operating under well-defined prevention procedures [9]. Over recent decades, two complementary approaches to HAI prevention have crystallised: a vertical (pathogen-targeted) strategy and a horizontal (hospital-wide standard precautions) strategy. The vertical approach focuses on reducing the risk posed by selected high-priority pathogens through active testing to identify asymptomatic carriers, isolation of colonised or infected patients, and where applicable—decolonisation. The horizontal approach aims to reduce infections caused by a broad range of microorganisms by strengthening HH and the use of personal protective equipment, environmental cleaning and disinfection, and promoting prudent antimicrobial use [10]. In practice, hospitals combine elements of both approaches according to local needs.

At CMHI, the IPC programme has evolved over many years as a combined strategy integrating horizontal measures with vertical pathogen-targeted interventions. An IPC Committee/Team has operated since the early 2000s, underpinned by formal policies for hygienic and surgical hand antisepsis, isolation/contact precautions, outbreak management, environmental cleaning and sterilisation, and routine surveillance of process and outcome indicators. In 2012, the HH programme was expanded and standardised through mandatory annual training (with practical components) delivered by WHO-certified instructors, a Polish-language instructional video incorporated into onboarding, direct-observation audits using WHO methodology, regular feedback to clinical units, and routine reporting to hospital leadership. In June 2013, following a sentinel *K. pneumoniae* ESBL(+) outbreak, CMHI formalised admission screening as a vertical risk-based intervention. Initial indications (transfers from other hospitals, previous CMHI hospitalisation and surgical admissions) were broadened in 2018 to include all surgical/invasive admissions and anyone hospitalised in any healthcare facility within the prior three months and updated again in 2022 to include foreign nationals. Each revision was aligned with evolving epidemiology and resource stewardship. Screening was operationalised alongside immediate contact precautions for carriers, reinforcing the horizontal platform (HH, environmental hygiene) with a vertical layer that identifies high-risk patients on entry. In parallel, AMS (established 2012) supported the platform through guideline-driven empiric therapy and peri-operative prophylaxis, formulary oversight, targeted audits, and hospital-wide monitoring of antibiotic use.

As admission screening at CMHI was broadened and embedded in routine practice, detection of colonisation by alert pathogens increased, whereas HAIs due to the same groups remained low and overall stable. This divergence is operationally coherent: admission screening increases case-finding by identifying colonised patients at entry, while hospital-wide standard precautions, particularly HH, contact precautions, and environmental hygiene, aim to limit onward transmission within the hospital. Importantly, colonisation in this study was assessed through admission screening, and therefore, the rising detection primarily reflects changes in screening intensity and case-finding strategy rather than acquisition during hospitalisation. Pathogen-specific patterns were consistent with this interpretation. ESBL-producing Enterobacterales showed the clearest rise in colonisation over time, but ESBL-related HAIs did not escalate, consistent with reports that early identification coupled with reliable horizontal measures suppress intra-hospital spread despite sustained importation pressure [11,12]. CPE appeared late and infrequently in screening, and only one CPE HAI occurred by study end mirroring paediatric centres where risk-based screening, applied on top of strong horizontal IPC, contains CPE without universal testing [13,14,15]. For MRSA and VRE, colonisation remained low overall (with VRE showing modest late increases), and corresponding HAIs were rare, aligning with the paediatric literature suggesting that adherence to standard precautions and timely isolation are more influential than MRSA decolonisation alone [16,17,18]. CAR-R were rarely detected in colonisation, and their HAI burden decreased in later years; although ecological data preclude causal inference, this pattern is compatible with sustained horizontal IPC operating in a setting with relatively stable transmission pressure [19,20,21,22]. Importantly, no routine oral decontamination or selective digestive decontamination protocols were used during the study period; therefore, the observed trends cannot be attributed to such strategies.

Interpretation of these findings must also consider changes in the hospital activity and care structure over time. During the study period, the estimated mean length of stay decreased substantially, reflecting a progressive shortening of average in-hospital exposure, a key determinant of cumulative HAI risk. At the same time, the proportion of patient-days attributable to intensive care (PICU and NICU combined) changed only gradually, without abrupt shifts in the overall care intensity. These indicators provide important contextual information for interpreting stable HAI incidence in an ecological analysis but cannot substitute for patient-level assessment of severity, device use, or other individual risk factors.

At CMHI, the ABHR use more than doubled over the study period, with a pronounced peak in 2020 and a partial return thereafter to levels still > 2 × the 2011 baseline. This trajectory is epidemiologically coherent. First, ABHR consumption is a pragmatic proxy for opportunity-based hand hygiene activity and often rises alongside programme maturation and feedback loops; multi-year hospital series report sustained increases in ABHR/1000 patient-days as hand-hygiene programmes embed [23,24]. Second, the 2020 spike mirrors the global COVID-19 effect: hospitals widely recorded surges in ABHR use and intensified attention to hand hygiene, followed by a partial reversion while remaining above pre-pandemic baselines [25,26]. Third, when set against European snapshots, CMHI’s post-pandemic volumes sit comfortably above typical reference ranges reported in national and EU exercises, which is consistent with the low and stable HAI burden observed despite the increasing detection of colonised carriers [2].

Two caveats are important for interpretation. ABHR consumption is a facility-level process indicator rather than a direct measure of moment-appropriate adherence; observed compliance (e.g., 75% in 2024) indicates residual room for improvement even under high ABHR availability. Still, longitudinal studies link higher ABHR use with stronger programme structure and, in some settings, reduced device- or unit-specific HAI rates, especially when embedded in multimodal bundles (training, direct observation, feedback, and environmental hygiene) [27]. In this context, the sustained elevation of ABHR use at CMHI should be interpreted as part of a broader horizontal IPC framework rather than as evidence of a direct causal effect on HAI outcomes.

Hospital-wide AMS activity at CMHI coincided with marked class-level rebalancing rather than a clear long-term fall in total J01. While the overall consumption in 2016–2024 fluctuated within ~278–356 DDD/1000 patient-days with no significant linear trend, consistent declines in carbapenems and fluoroquinolones and a sustained reduction in amoxicillin–clavulanate were observed, alongside increases in third- and fourth-generation cephalosporins and cefuroxime and moderate rises in piperacillin/tazobactam and glycopeptides. Such a substitution pattern is programmatically plausible: guideline-driven empiric choices, optimisation of peri-operative prophylaxis, formulary oversight, and targeted audits often manifest first as shifts within the antibiotic portfolio rather than as immediate reductions in the total consumption, especially in paediatrics, where DDD has recognised limitations as a metric, and DOT or indication-based measures are often preferred [28,29,30,31,32]. Interpretation of these trends should also consider concurrent changes in hospital throughput and length of stay, which may influence the exposure-adjusted consumption metrics.

The increase in antibiotic use observed around 2020 is consistent with early-pandemic reports of very high inpatient antibiotic prescribing for patients with COVID-19, driven by diagnostic uncertainty and precautionary practice; subsequent EU/EEA surveillance indicates that hospital antibiotic consumption patterns later normalised in many settings, albeit heterogeneously across countries [33,34]. Ecologically, two observations add contextual relevance. First, the downward pressure on carbapenem and fluoroquinolone use is consistent with reducing the selection for non-fermenters resistant to carbapenems; time-series and policy studies link constrained carbapenem/fluoroquinolone exposure with favourable resistance trajectories in *Pseudomonas* and other non-fermenters [35,36]. Second, the shift toward cephalosporins likely reflects deliberate AMS choices in empiric and targeted therapy. Overall, these findings suggest a redistribution of antibiotic class use consistent with stewardship objectives, while acknowledging that ecological data cannot demonstrate direct clinical impact.

### Strengths and Limitations

This fourteen-year hospital-wide analysis from a single tertiary paediatric centre uses stable HAI definitions and standardised denominators (per 1000 patient-days), reporting both process (admission screening intensity, ABHR consumption) and outcome measures (colonisation, HAI with alert pathogens, and antibiotic use by class). The analytic approach is transparent and consistent, combining linear trend analysis with assessment of monotonicity and highlights the central observation of increasing colonisation detection alongside persistently low HAI incidence. However, the ecological hospital-year design precludes causal inference and is susceptible to unmeasured confounding related to case-mix, occupancy, device use, and ward-level practice variation. The annual aggregation limits temporal resolution, and programme “exposures” were treated as time-varying institutional activities rather than quantified doses. To partially address concerns regarding hospital heterogeneity and changes in patient population, we incorporated hospital-level indicators of care structure, including the estimated mean length of stay and the proportion of patient-days attributable to intensive care, which provide contextual information on exposure duration and care intensity but do not substitute for patient-level risk stratification. Expanding screening inherently increases colonisation detection, complicating longitudinal comparisons. ABHR consumption is a proxy rather than a direct measure of opportunity-based hand-hygiene adherence, and analyses of organism-level resistance trends or patient-centred outcomes were beyond the scope of this study. Finally, DDD per 1000 patient-days has recognised limitations in paediatric populations and should be interpreted cautiously.

## 4. Materials and Methods

### 4.1. Setting and Population

The study was conducted at the Children’s Memorial Health Institute (CMHI), a tertiary multispecialty paediatric hospital (500 beds; including paediatric and neonatal intensive care units (PICU/NICU), oncology, surgery, transplantation, cardiac surgery, neurosurgery, and general paediatric wards).

This was a retrospective, observational, and hospital-wide ecological time-series study based on annual aggregated surveillance data. The unit of analysis was the hospital-year (2011–2024; for antibiotic consumption [ATC J01]: 2016–2024). No individual-level patient data were analysed, and no causal inference was attempted. The study evaluated temporal associations between the implementation/intensification of admission screening and hand-hygiene programmes and the operation of the AMS team and hospital-level outcomes (colonisation, HAI with alert pathogens, and antimicrobial use). Exposures were treated as time-varying institutional activities defined by programme timelines; no formal interrupted time-series modelling or seasonality adjustment was performed given the annual resolution of the data. The report follows the STROBE recommendations for observational studies, as applicable to ecological designs (Supplementary Table S1).

### 4.2. Definitions and Data Sources

Data on colonisations and HAIs came from the hospital surveillance system; HAI definitions and case classification remained unchanged throughout the study period. Alert pathogens included extended-spectrum β-lactamase-producing Enterobacterales (ESBL), carbapenemase-producing Enterobacterales (CPE), methicillin-resistant *Staphylococcus aureus* (MRSA), vancomycin-resistant *Enterococcus* (VRE), and carbapenem-resistant non-fermenting Gram-negative bacilli (CAR-R; i.e., *Pseudomonas aeruginosa* and *Acinetobacter baumannii*). Data on antibiotic and alcohol-based hand rub (ABHR) consumption came from the hospital pharmacy. High-selection-pressure antibiotics were defined as antimicrobial classes known to exert a particularly strong selective pressure for multidrug-resistant organisms, primarily carbapenems and fluoroquinolones, which were the main focus of stewardship-related analyses in this study. No imputation was applied for years with incomplete data; trends were presented graphically, and statistical tests were performed on the available observations.

The following measures and indicators were used:

process measures: (1) number of screening tests per 1000 patient-days; (2) consumption of ABHR in L/1000 patient-days;
outcome measures: (1) frequency of colonisation and HAI with alert pathogens per 1000 patient-days; (2) antibiotic consumption in defined daily doses (DDD)/1000 patient-days (overall and for selected groups).

### 4.3. Admission Screening and Laboratory Methods

Following an outbreak of *Klebsiella pneumoniae* ESBL(+) (April–June 2013; 24 patients: 7 infections and 17 colonisations), mandatory admission screening (nasal and rectal swabs) for alert pathogens (Enterobacterales ESBL, CPE, CAR-R, VRE, MRSA) was introduced in June 2013 for (1) patients admitted from other hospitals, (2) patients previously hospitalised at CMHI, and (3) patients scheduled for surgery. Previously, screening had been limited to patients admitted from other facilities.

In 2018, indications were expanded to include (a) all patients admitted for surgery or invasive procedures and (b) patients hospitalised in any healthcare facility (including long-term care) within the previous 3 months, regardless of the indication for the current admission. In 2022, all foreign nationals were additionally included.

Screening was performed by culturing nasal/throat/rectal swabs on chromogenic selective media for alert pathogens: ChromID MRSA, VRE, ESBL, CARBA, and OXA (bioMérieux, Marcy-l’Étoile, France). When colonies showed the characteristic growth described by the manufacturer, isolates were identified initially with the VITEK 2 system (bioMérieux, Marcy-l’Étoile, France) and, from 2017 onward, by matrix-assisted laser desorption/ionisation time-of-flight mass spectrometry (MALDI-TOF MS) using the Biotyper platform (Bruker Daltonics, Bremen, Germany).

Methicillin resistance in *S. aureus* was confirmed by disk diffusion with a 30 µg cefoxitin disk (BioMaxima S.A., Lublin, Poland). Vancomycin and teicoplanin resistance in *Enterococcus* spp. were determined by gradient diffusion strips to establish the minimum inhibitory concentrations (MICs) (bioMérieux, Marcy-l’Étoile, France). Antimicrobial susceptibility results were interpreted according to the current EUCAST (European Committee on Antimicrobial Susceptibility Testing) recommendations.

ESBL production in Enterobacterales was assessed using the double-disk synergy test (DDST) with disks containing cefotaxime 30 µg, ceftazidime 30 µg, cefepime 30 µg, and amoxicillin–clavulanate 20/10 µg (BioMaxima S.A., Lublin, Poland) [37].

Carbapenemase production in Enterobacterales was first investigated phenotypically using the disk diffusion–based tests recommended by the National Reference Centre for Antimicrobial Susceptibility (KORLD) [38,39,40]: an EDTA-based test for metallo-β-lactamases (MBL) [41], a boronic-acid test for KPC (Graso Biotech, Owidz, Poland) [42], and a 30 µg temocillin disk test for OXA-48-like enzymes (Oxoid Ltd., Basingstoke, UK) [43]. Since 2017, rapid confirmation has also used the NG-TEST CARBA-5 immunochromatographic cassette assay (NG Biotech, Guipry, France). Additional assays to detect carbapenemase activity in general included the CARBA NP test [44] and the CIM test [45].

For *Pseudomonas* and *Acinetobacter* spp., carbapenem susceptibility was determined by disk diffusion and interpreted per current EUCAST guidelines applicable at the time of testing; breakpoint revisions over the study period may have influenced the categorisation in long-term surveillance. Isolates non-susceptible to carbapenems were tested for carbapenemase production using the CARBA NP test for *Pseudomonas* or the CarbAcineto NP test [46] for *Acinetobacter* (ELITechGroup, Puteaux, France), as well as the CIM test. The enzyme class was then inferred phenotypically using EDTA and boronic-acid inhibition tests, as for Enterobacterales.

No routine oral decontamination or selective digestive decontamination protocols were implemented at hospital level during the study period.

### 4.4. HH Programme

Before 2012, ABHR at the point of care and educational materials (“5 moments of hand hygiene”) were already available; in 2012, the program was expanded and intensified (Polish translation of the NEJM hand hygiene video and inclusion in onboarding training for all newly hired staff, mandatory annual training delivered by WHO-certified specialists with practical components, direct observation audits according to the WHO methodology), with monitoring of ABHR consumption across all units. The results were reported annually to all staff and twice yearly to the Hospital Management and unit heads.

### 4.5. AMS

The AMS team was established in 2012 at CMHI. Activities included the following: (1) development and updating of local treatment guidelines and perioperative antibiotic prophylaxis (PAP); (2) on-demand therapeutic consultations; (3) formulary oversight with preauthorisation of selected antimicrobials; (4) annual AMS training; (5) twice-yearly audits of PAP adherence; (6) an annual retrospective audit of management of staphylococcal bacteraemia; (7) annual assessment of AMS compliance within the PPS survey; and (8) monitoring of antibiotic consumption by ATC groups across all units (systematically since 2016).

### 4.6. Statistical Analysis

Time-trend analyses were performed using annual data for 2011–2024 (for J01: 2016–2024). Linear regression was used, reporting the slope (average annual change) with 95% CI, R^2^, and *p*-value; monotonicity was assessed with Spearman’s rank correlation. Two-sided *p* < 0.05 was considered statistically significant. Statistical analysis was performed using SPSS version 20.0 (SPSS Inc., Chicago, IL, USA).

## 5. Conclusions

In this fourteen-year hospital-wide series from a tertiary paediatric centre, expansion of risk-based admission screening and sustained implementation of a multimodal HH programme were accompanied by the increased detection of colonisation with alert pathogens, while HAIs caused by the same groups remained low and overall stable. Over the same period, AMS coincided with marked redistribution of the antibiotic class use, characterised by sustained declines in carbapenems, fluoroquinolones, and amoxicillin–clavulanate, alongside increases in selected cephalosporins, without a clear long-term reduction in total antibiotic consumption.

Taken together, these descriptive hospital-level findings are consistent with a pragmatic approach to infection prevention and antimicrobial stewardship in tertiary paediatric care that combines risk-based admission screening, timely isolation and contact precautions, a durable horizontal IPC framework, and stewardship strategies aimed at limiting exposure to agents associated with high selection pressure. Given the ecological design, the results should be interpreted as hypothesis-generating. Future studies incorporating ward-level analyses, patient-level risk stratification, indication-adjusted antibiotic metrics (e.g., DOT), organism-specific resistance trends, and higher-resolution time-series methods are needed to refine attribution and quantify the programme effects.

## Data Availability

The data presented in this study are available on request from the corresponding author.

## Supplementary Materials

The following supporting information can be downloaded at https://www.mdpi.com/article/10.3390/antibiotics15020118/s1, Table S1: STROBE checklist; Table S2: Hospital admissions, patient-days, estimated length of stay, and intensive care utilisation at CMHI, 2011–2024.
