# Supplementary material for: From Screening to Outcomes: Fourteen-Year Hospital-Wide Surveillance of Alert Pathogens and Antimicrobial Use in a Paediatric Tertiary Hospital
Source: Antibiotics (Basel). 2026 Jan 26;15(2):118. doi: 10.3390/antibiotics15020118 (PMC12937373; doi:10.3390/antibiotics15020118)
Supplement: Supplementary file 1 [file antibiotics-15-00118-s001.zip › antibiotics-4084149-supplementary.pdf]

Supplementary Table S1. STROBE checklist

Item	Recommendation	Reported in manuscript (Section)
**1. Title and abstract**	Indicate the study design with a commonly used term	Title; Abstract
**2. Background/rationale**	Explain the scientific background and rationale	Introduction
**3. Objectives**	State specific objectives and hypotheses	Introduction
**4. Study design**	Present key elements of study design early	Materials and Methods (Study design)
**5. Setting**	Describe setting, locations, relevant dates	Materials and Methods (Setting and study period)
**6. Participants**	Eligibility criteria and selection methods	Materials and Methods (Study population; Screening strategy)
**7. Variables**	Clearly define outcomes, exposures, predictors	Materials and Methods (Definitions of colonisation, HAI, alert pathogens; Outcomes)
**8. Data sources/measurement**	Data sources and assessment methods	Materials and Methods (Microbiological data; IPC indicators; Pharmacy data)
**9. Bias**	Describe efforts to address potential sources of bias	Materials and Methods (Surveillance definitions; Consistent methodology); Discussion (Limitations)
**10. Study size**	Explain how study size was arrived at	Materials and Methods (Hospital-wide surveillance; Entire study population included)
**11. Quantitative variables**	Explain handling of quantitative variables	Materials and Methods (Incidence rates per 1,000 patient-days; ABHR metrics; DDD)
**12. Statistical methods**	Describe all statistical methods	Materials and Methods (Statistical analysis)
**12a. Subgroups/interactions**	Methods used to examine subgroups	Materials and Methods (Pathogen-specific analyses; Antibiotic classes)
**12b. Missing data**	Address missing data	Materials and Methods (Data completeness)
**12c. Loss to follow-up**	Address loss to follow-up	Not applicable (hospital-wide aggregated surveillance data)
**12d. Sensitivity analyses**	Describe sensitivity analyses	Not applicable. Formal sensitivity analyses were not performed due to the ecological design and annual aggregation of data; however, exploratory period-specific analyses (e.g., pandemic vs post-pandemic years) are reported descriptively in the Results and discussed in the Discussion.
**13. Participants**	Numbers at each stage of study	Materials and Methods (Hospital admissions; Screening tests); Results (Screening activity)
**14. Descriptive data**	Characteristics of study participants	Materials and Methods (Hospital characteristics); Results (Screening intensity; Patient-days)

15. Outcome data	Numbers of outcome events over time	Results (Colonisation and HAI incidence by pathogen)
**16. Main results**	Unadjusted estimates and precision	Results (All sections; Incidence trends with CIs, p-values)
**17. Other analyses**	Additional analyses	Results (Antibiotic consumption by class; Composite broad-spectrum pressure)
**18. Key results**	Summarise key results with reference to objectives	Discussion
**19. Limitations**	Discuss limitations and potential bias	Discussion (Limitations)
**20. Interpretation**	Cautious interpretation considering objectives and evidence	Discussion
**21. Generalisability**	Discuss external validity	Discussion
**22. Funding**	Source of funding and role of funders	Funding statement

Supplementary Table S2. Hospital admissions, patient-days, estimated length of stay, and intensive care utilisation at CMHI, 2011–2024

ICU includes combined paediatric intensive care units (PICU) and neonatal intensive care unit (NICU). Estimated mean length of stay (LOS) was calculated as the ratio of total patient-days to hospital admissions. ICU share represents the proportion of total hospital patient-days attributable to ICU care.

Year	Hospital admissions	Total patient-days	Estimated mean LOS (days)	ICU patient-days (PICU+NICU)	ICU share of total patient-days (%)
2011	37935	152024	4.01	16443	10.82
2012	37558	150646	4.01	17039	11.31
2013	40686	144187	3.54	17524	12.15
2014	39357	150239	3.82	18116	12.06
2015	42746	148515	3.47	18599	12.52
2016	41283	163243	3.95	22754	13.94
2017	40818	160003	3.92	24031	15.02
2018	41166	141241	3.43	22174	15.70
2019	41691	139844	3.35	21308	15.24
2020	30741	109250	3.55	18820	17.23
2021	38471	138528	3.60	19152	13.83
2022	41296	127076	3.08	18170	14.30
2023	45997	120863	2.63	18809	15.56
2024	51493	121285	2.36	18133	14.95
